# 3-Chloro-6-[2-(cyclo­pentyl­idene)hydrazin-1-yl]pyridazine

**DOI:** 10.1107/S1600536811011342

**Published:** 2011-03-31

**Authors:** Abdul Qayyum Ather, M. Nawaz Tahir, Misbahul Ain Khan, Muhammad Makshoof Athar

**Affiliations:** a Department of Chemistry, Islamia University, Bahawalpur, Pakistan; b Applied Chemistry Research Center, PCSIR Laboratories Complex, Lahore 54600, Pakistan; c Department of Physics, University of Sargodha, Sargodha, Pakistan; d Institute of Chemistry, University of the Punjab, Lahore, Pakistan

## Abstract

The asymmetric unit of the title compound, C_9_H_11_ClN_4_, contains two virtually planar mol­ecules that differ in conformation about the bond connecting the hydrazine and pyridazine units. The 3-chloro-6-hydrazinylpyridazine and cyclo­pentane groups are oriented at dihedral angles of 4.5 (3) and 8.8 (4)° in the two mol­ecules. In the crystal, the mol­ecules form a one dimensional polymeric structure extending along the *a* axis *via* N—H⋯N hydrogen bonds. The crystal stucired was an inversion twin [ratio of the twin domains = 0.73 (9):0.27 (9)].

## Related literature

For related structures, see: Ather *et al.* (2010**a* ▶,*b* ▶,c* ▶). For graph-set notation, see: Bernstein *et al.* (1995 ▶).
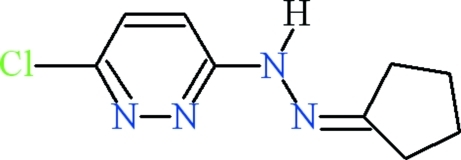


## Experimental

### 

#### Crystal data

C_9_H_11_ClN_4_
*M* *_r_* = 210.67
Orthorhombic, 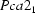

*a* = 10.180 (5) Å
*b* = 9.870 (5) Å
*c* = 20.049 (3) Å
*V* = 2014.5 (15) Å^3^
*Z* = 8
Mo *K*α radiation
μ = 0.34 mm^−1^
*T* = 296 K
0.30 × 0.15 × 0.14 mm

#### Data collection

Bruker Kappa APEXII CCD diffractometer
Absorption correction: multi-scan (*SADABS*; Bruker, 2005 ▶) *T*_min_ = 0.942, *T*_max_ = 0.950
7740 measured reflections
3355 independent reflections
2130 reflections with *I* > 2σ(*I*)
*R*_int_ = 0.044

#### Refinement

*R*[*F* ^2^ > 2σ(*F* ^2^)] = 0.050
*wR*(*F* ^2^) = 0.120
*S* = 1.00
3355 reflections
254 parameters
1 restraint
H-atom parameters constrained
Δρ_max_ = 0.18 e Å^−3^
Δρ_min_ = −0.17 e Å^−3^
Absolute structure: Flack (1983 ▶), 1307 Friedel pairs
Flack parameter: 0.73 (9)

### 

Data collection: *APEX2* (Bruker, 2009 ▶); cell refinement: *SAINT* (Bruker, 2009 ▶); data reduction: *SAINT*; program(s) used to solve structure: *SHELXS97* (Sheldrick, 2008 ▶); program(s) used to refine structure: *SHELXL97* (Sheldrick, 2008 ▶); molecular graphics: *ORTEP-3 for Windows* (Farrugia, 1997 ▶) and *PLATON* (Spek, 2009 ▶); software used to prepare material for publication: *WinGX* (Farrugia, 1999 ▶) and *PLATON*.

## Supplementary Material

Crystal structure: contains datablocks global, I. DOI: 10.1107/S1600536811011342/gk2360sup1.cif

Structure factors: contains datablocks I. DOI: 10.1107/S1600536811011342/gk2360Isup2.hkl

Additional supplementary materials:  crystallographic information; 3D view; checkCIF report

## Figures and Tables

**Table 1:** Hydrogen-bond geometry (Å, °)

*D*—H⋯*A*	*D*—H	H⋯*A*	*D*⋯*A*	*D*—H⋯*A*
N3—H3*A*⋯N5^i^	0.86	2.52	3.295 (5)	150
N3—H3*A*⋯N6^i^	0.86	2.27	3.088 (5)	159
N7—H7⋯N1^ii^	0.86	2.19	3.041 (5)	170

Symmetry codes: (i) 
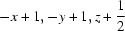
; (ii) 
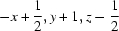
.

**Fig. 1.**
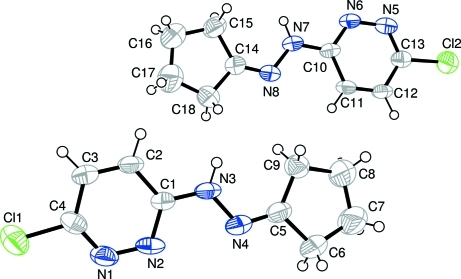
View of the title compound with the atom numbering scheme. The thermal ellipsoids are drawn at the 50% probability level. H-atoms are shown as small spheres of arbitrary radii.

**Fig. 2.**
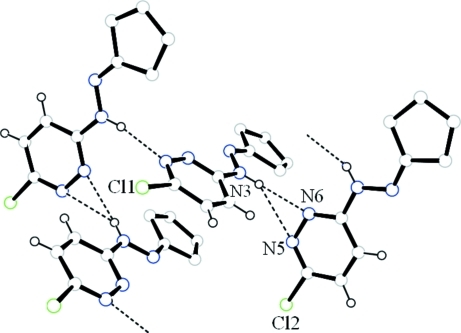
The partial packing diagram (PLATON; Spek, 2009) showing polymeric chains extending along the a-axis. The H-atoms of cyclopentane are omitted for clarity.

## supplementary crystallographic information

### Comment

In continuation to on our studies on 3-chloro-6-hydrazinylpyridazine derivatives (Ather *et al.*, 2010**a*,*b*,c*), the title compound (Fig. 1) is being reported here.

There are two symmetry independent molecule in the asymmetric unit of title compound that differ in conformation. In one molecule 3-chloro-6-hydrazinylpyridazine moiety A (C1—C4/N1—N4/CL1) and cyclopentane group B (C5–C9) are planar with r. m. s. deviations of 0.0104 and 0.0354 Å. The dihedral angle between A/B is 8.5 (4)°. In the second symmetry independent molecule 3-chloro-6-hydrazinylpyridazine moiety C (C10—C13/N5—N8/CL2) and cyclopentane gruop D (C14–C18) are also planar with r. m. s. deviations of 0.0068 and 0.0046 Å. The dihedral angle between C/D is 4.5 (3)°. The title compound consists of one dimensional polymeric chains via N–H···N hydrogen bonds extending along the crystallographic *a*-axis (Table 1, Fig. 2).

### Experimental

3-Chloro-6-hydrazinylpyridazine (0.5 g, 3.46 mmol), dissolved in ethanol (10 ml) and refluxed for 15 min. Cyclopentanone (0.291 g, 3.459 mmol) was added to the formar solution and refluxed about 3 h, till the completion of reaction monitored through TLC. On completion of the reaction mixture was concenterated under vacuum. The crude product was recrystallized in ethanol which yielded the light yellow needles of the title compound.

### Refinement

The structure was refined as an inversion twin with 0.73 (9):0.27((9) ratio of the twin domains. The H-atoms were positioned geometrically (N—H = 0.86, C–H = 0.93–0.97 Å) and were included in the refinement in the riding model approximation, with *U*_iso_(H) = x*U*_eq_(C, N), where x = 1.2 for all H-atoms.

### Crystal data

**Table:**

C_9_H_11_ClN_4_	*F*(000) = 880
*M**_r_* = 210.67	*D*_x_ = 1.389 Mg m^−^^3^
Orthorhombic, *P**c**a*2_1_	Mo *K*α radiation, λ = 0.71073 Å
Hall symbol: P 2c -2ac	Cell parameters from 1374 reflections
*a* = 10.180 (5) Å	θ = 2.9–28.3°
*b* = 9.870 (5) Å	µ = 0.34 mm^−^^1^
*c* = 20.049 (3) Å	*T* = 296 K
*V* = 2014.5 (15) Å^3^	Needle, light yellow
*Z* = 8	0.30 × 0.15 × 0.14 mm

### Data collection

**Table:**

Bruker Kappa APEXII CCD diffractometer	3355 independent reflections
Radiation source: fine-focus sealed tube	2130 reflections with *I* > 2σ(*I*)
graphite	*R*_int_ = 0.044
Detector resolution: 7.60 pixels mm^-1^	θ_max_ = 26.0°, θ_min_ = 2.9°
ω scans	*h* = −12→12
Absorption correction: multi-scan (*SADABS*; Bruker, 2005)	*k* = −12→11
*T*_min_ = 0.942, *T*_max_ = 0.950	*l* = −24→19
7740 measured reflections	

### Refinement

**Table:**

Refinement on *F*^2^	Secondary atom site location: difference Fourier map
Least-squares matrix: full	Hydrogen site location: inferred from neighbouring sites
*R*[*F*^2^ > 2σ(*F*^2^)] = 0.050	H-atom parameters constrained
*wR*(*F*^2^) = 0.120	*w* = 1/[σ^2^(*F*_o_^2^) + (0.053*P*)^2^] where *P* = (*F*_o_^2^ + 2*F*_c_^2^)/3
*S* = 1.00	(Δ/σ)_max_ < 0.001
3355 reflections	Δρ_max_ = 0.18 e Å^−^^3^
254 parameters	Δρ_min_ = −0.17 e Å^−^^3^
1 restraint	Absolute structure: Flack (1983), 1307 Friedel pairs
Primary atom site location: structure-invariant direct methods	Flack parameter: 0.73 (9)

### Special details

**Table:**

Geometry. All esds (except the esd in the dihedral angle between two l.s. planes) are estimated using the full covariance matrix. The cell esds are taken into account individually in the estimation of esds in distances, angles and torsion angles; correlations between esds in cell parameters are only used when they are defined by crystal symmetry. An approximate (isotropic) treatment of cell esds is used for estimating esds involving l.s. planes.
Refinement. Refinement of *F*^2^ against ALL reflections. The weighted *R*-factor *wR* and goodness of fit *S* are based on *F*^2^, conventional *R*-factors *R* are based on *F*, with *F* set to zero for negative *F*^2^. The threshold expression of *F*^2^ > σ(*F*^2^) is used only for calculating *R*-factors(gt) *etc*. and is not relevant to the choice of reflections for refinement. *R*-factors based on *F*^2^ are statistically about twice as large as those based on *F*, and *R*- factors based on ALL data will be even larger.

### Fractional atomic coordinates and isotropic or equivalent isotropic displacement parameters (Å^2^)

**Table:**

	*x*	*y*	*z*	*U*_iso_*/*U*_eq_	
Cl1	0.05268 (12)	−0.05719 (13)	0.64742 (7)	0.0730 (4)	
N1	0.2266 (4)	−0.1211 (3)	0.5585 (2)	0.0563 (10)	
N2	0.3221 (4)	−0.1007 (3)	0.5124 (2)	0.0540 (10)	
N3	0.4576 (4)	0.0479 (3)	0.4564 (2)	0.0564 (10)	
H3A	0.4848	0.1290	0.4488	0.068*	
N4	0.5125 (4)	−0.0589 (3)	0.4229 (2)	0.0576 (10)	
C1	0.3608 (4)	0.0263 (4)	0.5012 (2)	0.0403 (10)	
C2	0.3031 (4)	0.1383 (4)	0.5338 (2)	0.0495 (11)	
H2	0.3302	0.2261	0.5240	0.059*	
C3	0.2085 (4)	0.1165 (4)	0.5791 (2)	0.0489 (12)	
H3	0.1686	0.1871	0.6022	0.059*	
C4	0.1736 (4)	−0.0193 (4)	0.5896 (2)	0.0490 (12)	
C5	0.5972 (5)	−0.0292 (4)	0.3777 (3)	0.0553 (12)	
C6	0.6610 (5)	−0.1391 (5)	0.3385 (3)	0.0860 (17)	
H6A	0.7147	−0.1958	0.3671	0.103*	
H6B	0.5955	−0.1954	0.3169	0.103*	
C7	0.7435 (8)	−0.0690 (6)	0.2881 (4)	0.112 (3)	
H7A	0.8349	−0.0934	0.2945	0.134*	
H7B	0.7175	−0.0972	0.2436	0.134*	
C8	0.7283 (7)	0.0760 (6)	0.2945 (4)	0.101 (2)	
H8A	0.6859	0.1125	0.2551	0.121*	
H8B	0.8137	0.1187	0.2991	0.121*	
C9	0.6450 (4)	0.1042 (4)	0.3559 (2)	0.0574 (12)	
H9A	0.6972	0.1466	0.3906	0.069*	
H9B	0.5720	0.1634	0.3449	0.069*	
Cl2	0.72267 (11)	0.48307 (11)	−0.15057 (7)	0.0678 (4)	
N5	0.5643 (3)	0.6264 (3)	−0.07810 (19)	0.0544 (10)	
N6	0.4710 (3)	0.6489 (3)	−0.03214 (19)	0.0512 (9)	
N7	0.3193 (3)	0.5761 (3)	0.04280 (19)	0.0527 (10)	
H7	0.2969	0.6590	0.0495	0.063*	
N8	0.2597 (4)	0.4729 (3)	0.0777 (2)	0.0555 (11)	
C10	0.4144 (4)	0.5451 (4)	−0.0025 (2)	0.0444 (11)	
C11	0.4524 (4)	0.4099 (4)	−0.0162 (2)	0.0471 (11)	
H11	0.4124	0.3377	0.0057	0.056*	
C12	0.5459 (4)	0.3885 (4)	−0.0607 (2)	0.0466 (11)	
H12	0.5744	0.3014	−0.0710	0.056*	
C13	0.6001 (4)	0.5023 (4)	−0.0916 (2)	0.0464 (11)	
C14	0.1787 (4)	0.5052 (4)	0.1230 (3)	0.0533 (12)	
C15	0.1343 (4)	0.6416 (4)	0.1458 (3)	0.0658 (12)	
H15A	0.0928	0.6908	0.1096	0.079*	
H15B	0.2080	0.6943	0.1622	0.079*	
C16	0.0371 (6)	0.6142 (7)	0.2012 (3)	0.105 (2)	
H16A	0.0666	0.6569	0.2421	0.125*	
H16B	−0.0480	0.6515	0.1896	0.125*	
C17	0.0264 (7)	0.4674 (7)	0.2109 (4)	0.095 (2)	
H17A	0.0509	0.4442	0.2562	0.114*	
H17B	−0.0637	0.4389	0.2038	0.114*	
C18	0.1122 (5)	0.3979 (5)	0.1643 (3)	0.0870 (19)	
H18A	0.0617	0.3377	0.1359	0.104*	
H18B	0.1770	0.3446	0.1882	0.104*	

### Atomic displacement parameters (Å^2^)

**Table:**

	*U*^11^	*U*^22^	*U*^33^	*U*^12^	*U*^13^	*U*^23^
Cl1	0.0700 (7)	0.0779 (9)	0.0711 (9)	−0.0235 (6)	0.0072 (8)	0.0036 (8)
N1	0.068 (3)	0.0344 (19)	0.067 (3)	−0.0143 (19)	0.000 (2)	−0.0021 (19)
N2	0.069 (2)	0.0250 (17)	0.068 (3)	−0.0104 (17)	−0.008 (2)	−0.0068 (17)
N3	0.077 (3)	0.0236 (18)	0.068 (3)	−0.0011 (16)	0.011 (2)	−0.0014 (18)
N4	0.070 (3)	0.034 (2)	0.068 (3)	0.0026 (17)	0.005 (2)	−0.0077 (19)
C1	0.050 (3)	0.029 (2)	0.042 (3)	−0.0022 (17)	−0.005 (2)	−0.0016 (18)
C2	0.065 (3)	0.026 (2)	0.057 (3)	0.000 (2)	0.003 (3)	0.0006 (19)
C3	0.057 (3)	0.032 (2)	0.058 (3)	0.005 (2)	−0.006 (3)	−0.0003 (19)
C4	0.050 (3)	0.046 (3)	0.052 (3)	−0.014 (2)	−0.010 (2)	0.005 (2)
C5	0.065 (3)	0.039 (3)	0.062 (3)	0.008 (2)	0.003 (3)	−0.002 (2)
C6	0.100 (4)	0.048 (3)	0.110 (5)	0.008 (3)	0.035 (4)	−0.016 (3)
C7	0.149 (7)	0.072 (4)	0.114 (6)	0.012 (4)	0.069 (5)	−0.006 (4)
C8	0.117 (6)	0.069 (4)	0.119 (6)	0.008 (3)	0.056 (5)	0.010 (4)
C9	0.069 (3)	0.051 (3)	0.053 (3)	−0.003 (2)	0.007 (3)	0.000 (2)
Cl2	0.0699 (7)	0.0664 (8)	0.0672 (8)	−0.0042 (6)	0.0083 (7)	−0.0049 (7)
N5	0.066 (3)	0.035 (2)	0.063 (3)	−0.0066 (17)	−0.002 (2)	0.0023 (17)
N6	0.061 (2)	0.0271 (18)	0.066 (2)	−0.0021 (16)	0.001 (2)	−0.0045 (17)
N7	0.061 (2)	0.0283 (18)	0.069 (3)	0.0018 (17)	0.006 (2)	−0.0037 (18)
N8	0.063 (2)	0.033 (2)	0.070 (3)	−0.0065 (18)	0.010 (2)	0.0003 (18)
C10	0.053 (3)	0.028 (2)	0.052 (3)	0.0021 (18)	−0.005 (2)	−0.001 (2)
C11	0.055 (3)	0.025 (2)	0.062 (3)	−0.0033 (18)	0.001 (3)	0.003 (2)
C12	0.055 (3)	0.026 (2)	0.058 (3)	−0.0014 (19)	−0.004 (3)	−0.0035 (19)
C13	0.052 (2)	0.039 (2)	0.048 (3)	−0.002 (2)	−0.008 (2)	−0.002 (2)
C14	0.056 (3)	0.045 (3)	0.059 (3)	−0.001 (2)	0.002 (3)	−0.004 (2)
C15	0.071 (3)	0.056 (3)	0.071 (3)	0.005 (2)	0.001 (3)	−0.015 (3)
C16	0.121 (5)	0.075 (5)	0.117 (6)	0.010 (4)	0.041 (5)	−0.012 (4)
C17	0.108 (5)	0.097 (5)	0.079 (5)	0.003 (4)	0.017 (4)	−0.008 (4)
C18	0.105 (4)	0.057 (3)	0.098 (5)	−0.006 (3)	0.044 (4)	0.006 (3)

### Geometric parameters (Å, °)

**Table:**

Cl1—C4	1.732 (5)	Cl2—C13	1.730 (5)
N1—C4	1.299 (5)	N5—C13	1.306 (5)
N1—N2	1.357 (5)	N5—N6	1.342 (5)
N2—C1	1.333 (5)	N6—C10	1.317 (5)
N3—C1	1.351 (5)	N7—C10	1.362 (5)
N3—N4	1.370 (5)	N7—N8	1.377 (5)
N3—H3A	0.8600	N7—H7	0.8600
N4—C5	1.285 (6)	N8—C14	1.268 (5)
C1—C2	1.413 (5)	C10—C11	1.416 (5)
C2—C3	1.341 (6)	C11—C12	1.322 (6)
C2—H2	0.9300	C11—H11	0.9300
C3—C4	1.402 (6)	C12—C13	1.397 (6)
C3—H3	0.9300	C12—H12	0.9300
C5—C9	1.470 (6)	C14—C15	1.492 (6)
C5—C6	1.489 (6)	C14—C18	1.504 (7)
C6—C7	1.485 (8)	C15—C16	1.511 (7)
C6—H6A	0.9700	C15—H15A	0.9700
C6—H6B	0.9700	C15—H15B	0.9700
C7—C8	1.445 (7)	C16—C17	1.466 (9)
C7—H7A	0.9700	C16—H16A	0.9700
C7—H7B	0.9700	C16—H16B	0.9700
C8—C9	1.522 (8)	C17—C18	1.452 (8)
C8—H8A	0.9700	C17—H17A	0.9700
C8—H8B	0.9700	C17—H17B	0.9700
C9—H9A	0.9700	C18—H18A	0.9700
C9—H9B	0.9700	C18—H18B	0.9700
			
C4—N1—N2	120.7 (4)	C13—N5—N6	119.6 (3)
C1—N2—N1	117.7 (3)	C10—N6—N5	119.4 (3)
C1—N3—N4	120.1 (3)	C10—N7—N8	119.1 (3)
C1—N3—H3A	119.9	C10—N7—H7	120.5
N4—N3—H3A	119.9	N8—N7—H7	120.5
C5—N4—N3	116.3 (4)	C14—N8—N7	117.7 (4)
N2—C1—N3	118.4 (3)	N6—C10—N7	115.9 (4)
N2—C1—C2	122.4 (4)	N6—C10—C11	121.7 (4)
N3—C1—C2	119.2 (4)	N7—C10—C11	122.3 (4)
C3—C2—C1	119.1 (4)	C12—C11—C10	118.6 (4)
C3—C2—H2	120.5	C12—C11—H11	120.7
C1—C2—H2	120.5	C10—C11—H11	120.7
C2—C3—C4	115.9 (4)	C11—C12—C13	117.1 (4)
C2—C3—H3	122.0	C11—C12—H12	121.5
C4—C3—H3	122.0	C13—C12—H12	121.5
N1—C4—C3	124.2 (4)	N5—C13—C12	123.5 (4)
N1—C4—Cl1	116.7 (3)	N5—C13—Cl2	116.5 (3)
C3—C4—Cl1	119.1 (4)	C12—C13—Cl2	120.0 (3)
N4—C5—C9	129.5 (4)	N8—C14—C15	130.1 (4)
N4—C5—C6	119.9 (4)	N8—C14—C18	120.6 (4)
C9—C5—C6	110.6 (4)	C15—C14—C18	109.3 (4)
C7—C6—C5	105.4 (4)	C14—C15—C16	105.2 (4)
C7—C6—H6A	110.7	C14—C15—H15A	110.7
C5—C6—H6A	110.7	C16—C15—H15A	110.7
C7—C6—H6B	110.7	C14—C15—H15B	110.7
C5—C6—H6B	110.7	C16—C15—H15B	110.7
H6A—C6—H6B	108.8	H15A—C15—H15B	108.8
C8—C7—C6	109.9 (5)	C17—C16—C15	108.9 (5)
C8—C7—H7A	109.7	C17—C16—H16A	109.9
C6—C7—H7A	109.7	C15—C16—H16A	109.9
C8—C7—H7B	109.7	C17—C16—H16B	109.9
C6—C7—H7B	109.7	C15—C16—H16B	109.9
H7A—C7—H7B	108.2	H16A—C16—H16B	108.3
C7—C8—C9	108.2 (5)	C18—C17—C16	109.7 (5)
C7—C8—H8A	110.1	C18—C17—H17A	109.7
C9—C8—H8A	110.1	C16—C17—H17A	109.7
C7—C8—H8B	110.1	C18—C17—H17B	109.7
C9—C8—H8B	110.1	C16—C17—H17B	109.7
H8A—C8—H8B	108.4	H17A—C17—H17B	108.2
C5—C9—C8	105.1 (4)	C17—C18—C14	107.0 (5)
C5—C9—H9A	110.7	C17—C18—H18A	110.3
C8—C9—H9A	110.7	C14—C18—H18A	110.3
C5—C9—H9B	110.7	C17—C18—H18B	110.3
C8—C9—H9B	110.7	C14—C18—H18B	110.3
H9A—C9—H9B	108.8	H18A—C18—H18B	108.6
			
C4—N1—N2—C1	1.3 (6)	C13—N5—N6—C10	−1.8 (6)
C1—N3—N4—C5	175.2 (4)	C10—N7—N8—C14	174.9 (4)
N1—N2—C1—N3	178.8 (4)	N5—N6—C10—N7	−179.1 (3)
N1—N2—C1—C2	−2.2 (6)	N5—N6—C10—C11	2.2 (6)
N4—N3—C1—N2	0.8 (6)	N8—N7—C10—N6	−177.4 (4)
N4—N3—C1—C2	−178.2 (4)	N8—N7—C10—C11	1.4 (6)
N2—C1—C2—C3	2.1 (6)	N6—C10—C11—C12	−1.0 (6)
N3—C1—C2—C3	−178.9 (4)	N7—C10—C11—C12	−179.7 (4)
C1—C2—C3—C4	−0.9 (6)	C10—C11—C12—C13	−0.4 (6)
N2—N1—C4—C3	−0.1 (6)	N6—N5—C13—C12	0.2 (6)
N2—N1—C4—Cl1	−180.0 (3)	N6—N5—C13—Cl2	−179.1 (3)
C2—C3—C4—N1	0.0 (6)	C11—C12—C13—N5	0.9 (6)
C2—C3—C4—Cl1	179.8 (3)	C11—C12—C13—Cl2	−179.8 (3)
N3—N4—C5—C9	−0.2 (7)	N7—N8—C14—C15	0.4 (7)
N3—N4—C5—C6	−179.5 (4)	N7—N8—C14—C18	−178.5 (4)
N4—C5—C6—C7	175.6 (6)	N8—C14—C15—C16	179.9 (5)
C9—C5—C6—C7	−3.9 (7)	C18—C14—C15—C16	−1.1 (6)
C5—C6—C7—C8	−1.7 (8)	C14—C15—C16—C17	0.7 (6)
C6—C7—C8—C9	6.3 (9)	C15—C16—C17—C18	0.0 (8)
N4—C5—C9—C8	−171.9 (6)	C16—C17—C18—C14	−0.6 (7)
C6—C5—C9—C8	7.5 (6)	N8—C14—C18—C17	−179.8 (5)
C7—C8—C9—C5	−8.4 (7)	C15—C14—C18—C17	1.1 (6)

### Hydrogen-bond geometry (Å, °)

**Table:**

*D*—H···*A*	*D*—H	H···*A*	*D*···*A*	*D*—H···*A*
C2—H2···N5^i^	0.93	2.73	3.500 (6)	141
N3—H3A···N5^i^	0.86	2.52	3.295 (5)	150
N3—H3A···N6^i^	0.86	2.27	3.088 (5)	159
N7—H7···N1^ii^	0.86	2.19	3.041 (5)	170
C12—H12···N4^iii^	0.93	2.55	3.323 (5)	140

Symmetry codes: (i) −*x*+1, −*y*+1, *z*+1/2; (ii) −*x*+1/2, *y*+1, *z*−1/2; (iii) −*x*+1, −*y*, *z*−1/2.

## References

[bb1] Ather, A. Q., Tahir, M. N., Khan, M. A. & Athar, M. M. (2010*a*). *Acta Cryst.* E**66**, o2107. 10.1107/S1600536810024402 PMC3007326 21588398

[bb2] Ather, A. Q., Tahir, M. N., Khan, M. A. & Athar, M. M. (2010*b*). *Acta Cryst.* E**66**, o2441. 10.1107/S1600536810034239 PMC3008033 21588763

[bb3] Ather, A. Q., Tahir, M. N., Khan, M. A. & Athar, M. M. (2010*c*). *Acta Cryst.* E**66**, o2499. 10.1107/S160053681003504X PMC2983409 21587496

[bb4] Bernstein, J., Davis, R. E., Shimoni, L. & Chang, N.-L. (1995). *Angew. Chem. Int.* Ed. Engl. **34**, 1555–1573.

[bb5] Bruker (2005). *SADABS* Bruker AXS Inc., Madison, Wisconsin, USA.

[bb6] Bruker (2009). *APEX2* and *SAINT* Bruker AXS Inc., Madison, Wisconsin, USA.

[bb7] Farrugia, L. J. (1997). *J. Appl. Cryst.* **30**, 565.

[bb8] Farrugia, L. J. (1999). *J. Appl. Cryst.* **32**, 837–838.

[bb9] Flack, H. D. (1983). *Acta Cryst.* A**39**, 876–881.

[bb10] Sheldrick, G. M. (2008). *Acta Cryst.* A**64**, 112–122. 10.1107/S0108767307043930 18156677

[bb11] Spek, A. L. (2009). *Acta Cryst.* D**65**, 148–155. 10.1107/S090744490804362X PMC2631630 19171970

